# Can EEG Be Adopted as a Neuroscience Reference for Assessing Software Programmers’ Cognitive Load?

**DOI:** 10.3390/s21072338

**Published:** 2021-03-27

**Authors:** Júlio Medeiros, Ricardo Couceiro, Gonçalo Duarte, João Durães, João Castelhano, Catarina Duarte, Miguel Castelo-Branco, Henrique Madeira, Paulo de Carvalho, César Teixeira

**Affiliations:** 1 Department of Informatics Engineering, CISUC-Centre for Informatics and Systems of the University of Coimbra, University of Coimbra, P-3030-790 Coimbra, Portugal; rcouceir@dei.uc.pt (R.C.); duarte.1995@live.com.pt (G.D.); jduraes@isec.pt (J.D.); henrique@dei.uc.pt (H.M.); carvalho@dei.uc.pt (P.d.C.); cteixei@dei.uc.pt (C.T.); 2 Coimbra Polytechnic—ISEC, R. Pedro Nunes, P-3030-199 Coimbra, Portugal; 3 ICNAS-Institute of Nuclear Sciences Applied to Health, University of Coimbra, P-3000-548 Coimbra, Portugal; joaocastelhano@uc.pt (J.C.); catarinaduarte@uc.pt (C.D.); mcbranco@fmed.uc.pt (M.C.-B.); 4 CIBIT-Coimbra Institute for Biomedical Imaging and Translational Research, University of Coimbra, P-3000-548 Coimbra, Portugal

**Keywords:** software engineering, bio-signal processing, electroencephalogram, biofeedback, human error

## Abstract

An emergent research area in software engineering and software reliability is the use of wearable biosensors to monitor the cognitive state of software developers during software development tasks. The goal is to gather physiologic manifestations that can be linked to error-prone scenarios related to programmers’ cognitive states. In this paper we investigate whether electroencephalography (EEG) can be applied to accurately identify programmers’ cognitive load associated with the comprehension of code with different complexity levels. Therefore, a controlled experiment involving 26 programmers was carried. We found that features related to Theta, Alpha, and Beta brain waves have the highest discriminative power, allowing the identification of code lines and demanding higher mental effort. The EEG results reveal evidence of mental effort saturation as code complexity increases. Conversely, the classic software complexity metrics do not accurately represent the mental effort involved in code comprehension. Finally, EEG is proposed as a reference, in particular, the combination of EEG with eye tracking information allows for an accurate identification of code lines that correspond to peaks of cognitive load, providing a reference to help in the future evaluation of the space and time accuracy of programmers’ cognitive state monitored using wearable devices compatible with software development activities.

## 1. Introduction

Software defects (i.e., bugs) represent the most enduring problem of software quality. In spite of decades of research and advances in software engineering and software reliability, the number of defects per 1000 lines of delivered code (KLoC) remains astonishingly high. Steve McConnell’s seminal book [1] points to an industry average of about 15 defects per KLoCs, which is a very large standard deviation. Even when software is developed using highly mature processes, the deployed code still has a relatively high defect density, from 1 to 5 bugs per KLoC [2,3,4,5]. More recent field data from several projects show defects densities from 1 to 6 bugs per KLoC [6], suggesting that in real projects, often characterized by millions of lines of code, the defect density remains quite significant and represents a huge cost for the software industry and society in general. Subsequently, reports on the finance impact of software defects and software failures show dramatic figures. The global cost of debugging software defects was estimated as $312 billion in 2013 [7], and the cost of software failures for the worldwide economy reached $1.1 trillion in 2016 [8] while “software failures affected 3.6 billion people in 2017, causing US$1.7 trillion in financial losses” [9].

Known causes of human error from a software developer perspective such as fatigue, cognitive overload, attention slips, and stress have not been much addressed in software engineering research. All these cognitive states (high mental effort, stress level, attention shifts, cognitive overload, mental fatigue) have been associated with error-prone scenarios, as established by human error models [10] and their adaptation to software development tasks [11,12], but so far none of the available software development methodologies uses any form of direct information from the cognitive state of the software developer.

The idea of using biometric measures such as heart rate variability (HRV), respiratory responses, pupillary response (pupillometry), electrodermal activity (EDA), or electromyogram (EMG) to gather information on the cognitive load while carrying out specific tasks (and inherently infer the difficulty associated with such tasks) is not new [13,14,15,16]. Furthermore, specific biometric measures such as EDA have also been proposed to discriminate stress from cognitive load [17,18]. However, only in recent years has the use of biometric measures been proposed for the context of software development. First works have shown that biometric measures can be linked to task difficulty or difficulty in comprehending code snippets [19], and that HRV can be used to predict the quality of the code made by programmers, which is useful to optimizing software testing [20].

Recent studies proposed a broader idea named **Biofeedback Augmented Software Engineering** [21] to integrate biometric measures such as HRV and pupillometry [22] in the software development process through the use of **cognitive state code annotations** [23]. The proposal consists of using eye tracking devices, particularly non-intrusive desktop eye trackers, to provide accurate information on the code lines where the programmer is looking, and annotate such code lines with the programmer’s cognitive state (expressing cognitive load, distractions, fatigue, etc.) gathered using HRV and pupillometry [23]. Cognitive state code annotations are expected to allow predictions of code quality in order to provide online warnings to the programmer, or to guide code inspections or the software testing effort in general. The idea of such predictions is intuitively simple and can be illustrated by straightforward examples (just three examples among many others):

- If the annotations associated with a given set of code lines say that the programmer is under high cognitive overload while producing/reviewing such code lines, then the chance of having software bugs in such code lines is higher than normal;
- If the code is complex (measured by classic complexity metrics) and the annotations show that the programmer is distracted while dealing with such code lines, then the probability of software bugs in the annotated code lines is higher than normal;
- If the code is simple and the annotations show that the programmer is under high mental effort, then the probability of software bugs in the annotated code lines is higher than normal.

Importantly, these first studies [19,20,21,22,23] have shown that programmers’ cognitive load during typical code development activities can be assessed using biometric measures obtained through wearable and low intrusive devices compatible with standard software development environments. Although quite encouraging, all these recent studies [19,20,21,22,23] have used wearable technology to infer programmer’s cognitive states while carrying out software development tasks, and all of them share an inherent limitation: The programmers’ cognitive states are inferred indirectly, through peripheral physiological signals driven by the autonomic nervous system (ANS) (e.g., HRV, pupillometry, EDA, EMG, among others), which raises the question of the accuracy and precision of the cognitive states assessed in that way. Furthermore, the response time of the ANS driven signals captured by wearable technology is another important concern, as the whole idea of cognitive code line annotation requires a timely response (or at least the delay must be known) to achieve accurate annotations. And, finally, physiological responses driven by the ANS is influenced by many other stimuli, which means that the signals gathered by the wearable devices will include noise and spurious physiological reactions not related to the software development activities.

There are other alternative technologies that do not focus on the recordings of ANS-related biosignals to assess the cognitive states of a subject, such as face analysis or gaze analysis. However, to the best of our knowledge, most studies exploring face analysis to assess cognitive states use it alongside other biosignasl [24]. Furthermore, for face analysis, the recording of video or images of the software programmers, while they are performing software tasks, can be psychologically intrusive. Regarding gaze analysis, there are a lot of research on this topic in software engineering [25], but most of the works focus on the afterwards analysis, i.e., based on the fixations, saccades, and scan-paths, needing some time to obtain the overall analysis of the gaze points. Consequently, it presents a low response time to gather information about the complexity of the task and inherent cognitive load. Therefore these type of approach might be useful as an complementary analysis, but not to validate the accuracy and precision of the cognitive states assessed based on physiological signals driven by the ANS.

These important limitations of all the existing proposals towards the use of biometric measures to improve the software quality can only be addressed through the creation of a ground truth to evaluate the accuracy of wearable devices that rely on physiologic manifestations driven by the ANS. This paper evaluates existing and new electroencephalography (EEG) biomarkers related to the central nervous system (CNS), sensitive to different levels of cognitive load in the context of software development.

EEG can be a powerful tool to this end, since it records the electrical activity of the brain, which is one of the most relevant physiological areas analyzed when assessing cognitive states [26]. From this signal we can explore and extract different and useful information to infer directly the cognitive states of the subject from the brain activity recorded, and still offers a less intrusive approach than other imaging techniques such as functional magnetic resonance imaging (fMRI) and near field infrared spectroscopy (fNIRS). Therefore, with our experiment, we intend to investigate and respond to the following research goals: (i) The cognitive load measured using EEG is representative of the subjective perception of code complexity; (ii) the complexity measured using classic software complexity metrics do not translate the EEG and subjective perception results from the code snippets; and (iii) EEG biomarkers combined with eye-tracking data can differentiate code regions that cause a higher cognitive load. Based on those hypotheses, this paper proposes the use of EEG to create a ground truth to be used as a reference in the evaluation of the accuracy of wearable devices. Although EEG is turning into a low cost and portable solution with advances in technology [27,28], it still cannot be used in normal software development environments, because it is too intrusive (in most practical realizations, it needs a cap to apply the large number of electrodes to the subject).

In short, the contributions of this paper are the following:

- Proposes the very first approach to use EEG as a reference to evaluate and improve the accuracy of wearable devices that rely on physiologic manifestations driven by the ANS, as indicators of programmers’ cognitive load. This contribution includes the online availability of a package including the proposed method, protocols, datasets, and EEG results;
- Demonstrates the viability of using EEG-derived features for assessing the cognitive load of programmers in comprehending program snippets of different complexities;
- Shows that cognitive load of programmers in code comprehension is consistent with the subjective perception of the programmers on the complexity and mental effort spent on comprehending a given program snippet;
- Confirms that software complexity metrics have clear limitations as indicators of the code complexity as perceived by (human) programmers, and shows that programmers’ perceived code complexity saturates very quickly as complexity metrics increase. This contribution has a relevant impact on existing software development practices, as it shows that the use of complexity metrics by the software industry (as a code complexity indicator during development) must be revisited to include both complexity metrics and programmers’ perceived code complexity measures.

The next section presents additional background and related work, particularly on cognitive state assessment using EEG. Section 3 describes the controlled experiment, the data, the acquisition protocol, and also details the methods used for preprocessing EEG data, feature engineering, and classification. Section 4 presents the main results and respective discussion, and proposes the use of the EEG as a reference to calibrate wearable devices that collect physiological signals driven by the ANS. Section 5 presents the threats to the validity of this work and Section 6 concludes the paper.

## 2. Background and Related Work

The root causes of software defects are buried deep in human error manifestations and must be addressed in an interdisciplinary perspective involving software engineering, cognitive psychology, neuroscience, and even biomedical fields. In fact, this interdisciplinary research perspective on software defects has gained ground and is now an emergent research line. The next subsections briefly overview the key aspects of this interdisciplinary research effort.

### 2.1. Software Complexity Metrics and Software Quality

Software complexity metrics are used worldwide in software engineering to guide software testing coverage, to estimate software defect density, and even to determine the most adequate component granularity in software architectures. In general, it is assumed that complexity metrics also portray the inherent complexity of software artefacts, as perceived by (human) software developers. However, it is known that complexity metrics deviate considerably from human perceived complexity in code structures such as recursive or multi-threading programming, as in these cases the complexity is not in the code structures (usually very compact) but in the recursive and/or parallel nature of the code. In addition, recent work has exposed the limits of complexity metrics as a tool to express software complexity in the human/programmers sense [21]. The present paper reinforces this conclusion using the much more reliable measurement provided by the EEG. In fact, as shown in Section 4, subjective complexity felt by participants (measured by the NASA TLX tool [29]) is inline with the cognitive load measured by EEG results. However, both measurements (i.e., EEG and subjective complexity results) deviate considerably from software complexity metrics, showing that the use of complexity metrics as predictors of a programmer/tester’s cognitive effort (and, consequently, predictor of error prone code) could be misleading.

Metrics can be relative to the code, documentation, or programmer subject [30]. Classic code-related metrics (i.e., McCabe [31] and Halstead’s [32] metrics) focus mainly on code and data structures. Well-known examples of code metrics include McCabe Cyclomatic Complexity (V(G)) [31], average Number of Nested Block Depth, Number of Parameters and Lines of codes, among others [33]. Documentation-related metrics are used to measure the quality of the code documentation, while the developer-related metrics are used to quantify the programmer’s experience, such as the time (in years) spent programming in general or/and in a specific programming language. A brief description of the metrics used for code complexity and comprehensibility assessment [34], that are mentioned throughout this paper, are presented in Table 1.

### 2.2. Electroencephalography

Code comprehension has a key role in the software development process. An extensive experimental study [35] shows that programmers spent about 70% time in code comprehension tasks, while writing software code. This central role of code comprehension maybe explains the recent interest of the neuroscience community in researching the brain and neural mechanisms related to programming tasks, especially code comprehension. Most of these neuroscience studies rely on complex and intrusive imaging equipment such as functional magnetic resonance imaging (fMRI) [36,37,38,39,40,41,42,43] and near field infrared spectroscopy (fNIRS) [44,45]. In this section we will provide a brief background of EEG and discuss related work on EEG during software development task, due to its relevance for this paper.

EEG can be a powerful tool to help understanding the brain mechanisms behind code comprehension and subsequently the complexity associated with software codes and human error while coding. The scalp-EEG is the most frequently used, since it is non-invasive and it provides a general perspective of the brain to understand the neural mechanisms [46]. At the scalp level, the signals recorded with EEG are the result of the activity of populations of pyramidal neurons that when fired in a synchronized way produce potential differences that cross bone and skin and can be sensed externally in the microvolts (μV) range. For interpretation, the EEG signal is commonly divided into 5 main frequency bands (Delta, Theta, Alpha, Beta, and Gamma) [47], presented in Table 2. It should be noted that this is just a simplified idea because there are subtypes of Alpha and Theta bands, for example.

Several research studies reporting the analysis of general task engagement and mental workload through the acquisition of EEG have been published in the last 20 years and in an extensive range of fields [48,49,50]. In many of them, strong correlations were found between the cognitive states of the subjects and the EEG frequency bands. The findings pointed out mainly to the strength of Theta and Alpha waves to represent these mental states [49,50,51,52].

Fritz et al. [19] accomplished the first automated approach for the assessment of a mental workload during code comprehension using psycho-physiological information (eye-tracker, EEG, and EDA). With data recorded from 15 software programmers, the authors achieved a F-measure (harmonic mean of the precision and recall) of only 56.73% using EEG information, which increased up to 67.71% by fusing the information from the three sensors.

Igor Crk and Timotthy Kluthe [53] performed a study reporting a binary qualification of a programmer’s expertise through code comprehension using recorded EEG signals from 14 electrodes in 34 participants. By using information of Theta and Alpha bands, the authors could distinguish the expertise with an accuracy from 53% to 63% depending on the task. Later, Lee et al. [54] also recorded EEG signals, using 13 electrodes, from 18 subjects, to analyze neurophysiological processes occurring during code comprehension tasks and the possibility to distinguish between expert programmers from beginners. The results showed that high frequencies are dominant features, namely Beta and Gamma bands, and the most significant channels were from frontal and parietal regions.

Lee et al. [55] conducted a study involving 38 participants with the aim of predicting their expertise level (novice or expert) or the difficulty (easy/difficult) of the comprehension tasks they performed, through the use of EEG and eye tracking data. The best result obtained for the prediction study of the difficulty of the task was a F-Measure of 66.6%, while for the study of prediction of the participants’ level of expertise, a F-Measure of 97% was achieved. Although striking results have been obtained, the authors did not specify what features were extracted from the recorded EEG, keeping unknown the EEG features that led to these surprising results.

Yeh et al. [56] carried out a controlled experiment in order to investigate the brain activity of 8 participants while they were performing code comprehension tasks of 12 confusing or non-confusing code snippets. Using only eight EEG electrodes from the frontal region of the brain, the authors computed the power of the Theta and Alpha bands of the signals, and found for all eight participants, there were significant statistical differences between the two categories of code snippets. Nevertheless, there were no significant statistical differences between the codes of the same category using such EEG features.

The most recent study on this topic was conducted by Kosti et al. [57] in 2018 and had the purpose of investigating the brain activity during two different programming tasks: Comprehension and inspection of syntax errors in C code. Using only 14 electrodes, they recorded EEG signals from 10 participants. The authors found that Theta, Beta, and Gamma waves during comprehension tasks correlate with cognitive effort, with stronger correlations observed for higher frequency waves. Another surprising finding was the detection of a much higher activation of Theta and Beta waves in comprehension tasks than in tasks of inspection of syntax errors. Authors argued that this was due to the fact of inspection tasks being considered easier tasks that do not require as much effort in the imagination of the program output as it is demanded for comprehension tasks.

Despite the encouraging results, most of the authors are aware of the limitations of such results, either because of the low number of participants or given that there are few studies in this area, making it difficult to make a comparison of results and conclusions. Furthermore, the studies that presented high performance did not indicate the most discriminant features for further investigation of their feasibility as potential biomarkers, which is a significant challenge for reproducing these results. However, such achievements prompt a further investigation of biosignals such as EEG, which, given the current technological advances and the existence of low-cost off-the-shell acquisition devices, are becoming more user friendly [27,58]. Furthermore, it can also encourage the investigation of correlations between EEG biomarkers with the ANS responses, in order to explore the possibility of replacing EEG by more comfortable and non-intrusive measurement modalities that allow monitoring of such responses in daily life conditions.

The limited number of studies and the small number of participants make it difficult to establish measures regarding cognitive load in Software Engineering. Our study differs from previous ones by providing a systematic analysis using EEG information with a considerable number of participants and having control elements such as the use of the NASA TLX tool to assess the subjective complexity of the code, as perceived by participants. The main aim is to understand the brain activity involved during different types of tasks and propose the use of EEG as a reference to attest the accuracy of the programmers’ cognitive load code annotation using simple, non-intrusive, and wearable biosensors that rely only on physiologic manifestations driven by the ANS (and not on direct brain signals such as EEG).

## 3. Materials and Methods

### 3.1. Protocol

**Figure 1:**
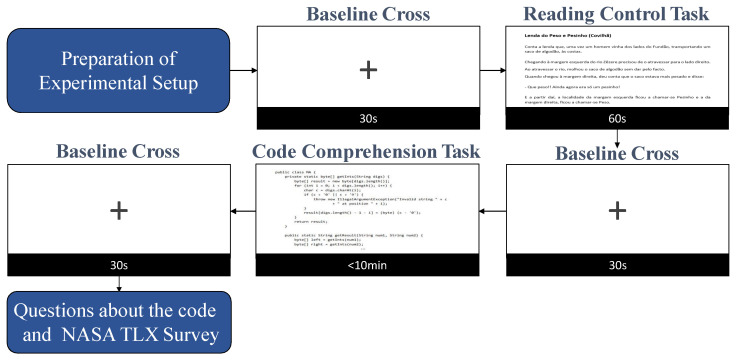
Representative schematics of one trial procedure, involving the fixed cross, in a screen, before and after the relevant tasks for analysis, i.e., the reading control task and the code comprehension task.

Subjects were submitted to three different trials of code comprehension tasks using three code snippets written in Java. The three code snippets, named as Code 1, Code 2, and Code 3, have different complexity levels, being Code 1 the simplest code and Code 3 the most complex one. Each trial consisted of a Control task of text reading in natural language (60 s maximum) and a task of code comprehension (10 min maximum). Before and after each task, a screen with a cross in the middle was shown for 30 s to the subject, acting as a baseline interval for the next task (see Figure 1).

After each trial, the subjects answered two questionnaires. In the first one, the subject had to explain the purpose of the code and the general idea of the algorithm/code structure with the goal of knowing whether the participant has understood the code or not. In reality, a second, and very important goal of the first questionnaire was to create an incentive for the participant to really try to understand the code, as the participant was informed beforehand that he/she would be asked about the code at the end of the trial. In the second questionnaire, the subject filled a survey based on NASA-TLX (Task-Load Index) survey [59] with four questions, rating it from 1 to 6, in order to assess the subjective mental effort, task fulfilment, pressure over time, and frustration of the subject while doing the code comprehension task.

The acquisition protocol is represented in Figure 1 with an estimated experience time of about one and a half hours for each subject—around 45 min for the preparation of experimental setup and then 45 min for the trial procedures.

**Figure 2:**
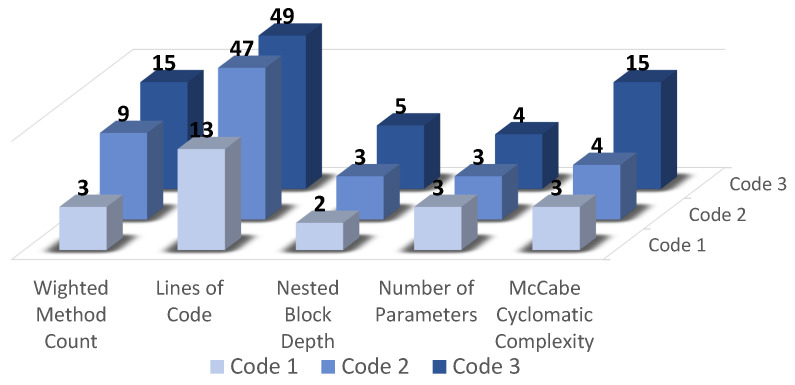
Complexity level of each code tasks according to each one of the five software complexity metrics used.

The order of the trials is always the same (i.e., no randomization) to assure that all the code comprehension tasks were executed in the same conditions by all the participants. The participants were not informed about the complexity of each program to avoid bias. The order used was Code 1 → Code 2 → Code 3, being Code 1 the less complex and Code 3 the most complex, at least according to software complexity metrics (as we will see in the results, participants considered Code 2 and Code 3 as having similar complexity). Figure 2 shows the code complexity metrics of the three programs used in the study. According to the metrics, differences between the three different codes complexity are visible, being the major difference noticed for the McCabe Cyclomatic Complexity metric, one of the most popular and widely used in software development [60].

Our dataset, experiment protocol and the sample programs (Code 1, Code 2, and Code3) are publicly available in the repository of the H2020 project AI4EI (A European AI On Demand Platform and Ecosystem) in the following link: https://ai4eu.dei.uc.pt/base-mental-effort-monitoring-dataset (accessed on 15 March 2021).

### 3.2. Participants, Acquisition Setup, and Quality Control

EEG signals were recorded from 30 subjects that were selected after a series of interviews. Subjects were students, university professors, and professional software developers, with experience in Java programming language. Specifically, from the 30 participants, 24 were male and 6 female, with ages ranging from 19 to 42, and average age of 24 years old. In addition, through the interview and based on years of experience in Java programming or in the number of lines of code programmed in Java in the last months or years, the participants were classified into three levels of proficiency: Intermediate, advanced, or expert, including 13 intermediate, 12 advanced, and 5 expert participants.

EEG signals were acquired using the Neuroscan SynAmps 2 amplifier, from Compumedics, at a sampling frequency of 1000 Hz, with 64 channels placed according to the International 10-10 system. Neuroscan also included four integrated bipolar leads for EMG, ECG, and the ocular-movement references VEOG (vertical electrooculogram) and HEOG (horizontal electrooculogram).

**Figure 3:**
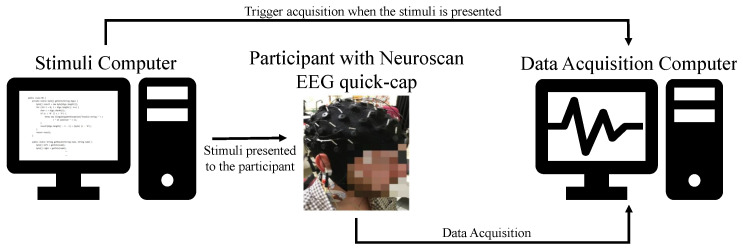
Schematic representation of the acquisition setup.

In the acquisition setup designed to record the EEG data, the EEG quick-cap was connected to the amplifier through the EEG HeadBox, which was connected to the acquisition computer that controls the whole experience, communicating synchronously with all sensors and to a second computer, used to present the stimuli to the participant and, additionally, sends the trigger to the acquisition computer (see Figure 3).

During the data acquisition of one of the subjects, several electrodes in relevant locations did not properly work, therefore this subject was discarded in posterior analysis. In addition, three more subjects were later removed since after analyzing the eye tracking data, those participants were more than 90% of the time out of the areas of interest (the codes), suggesting that they did not really tried to comprehend the code during some of the trials. Thus, the initial dataset was reduced to 26 subjects.

The data collection was authorized by all the participants involved and the written consent was approved by the Ethics Committee of the Faculty of Medicine of the University of Coimbra, in accordance with the Declaration of Helsinki.

### 3.3. Preprocessing

The step of preprocessing is mandatory for cleaning as much as possible the EEG data, yet preserving the neural content, in order to get a reliable analysis and interpretation of the postprocessed neural signals. This step was performed using the open source toolbox EEGLAB [61], one of the most widely used software for preprocessing/analysis of EEG data. Before proceeding to filtering, the EEG channels location information was added to the data, and the non-used channels, i.e., the mastoid electrodes (M1, M2) and the cerebellar electrodes (CB1, CB2), were removed.

#### 3.3.1. Filtering

Filtering followed the usual pre-processing applied in EEG processing. In particular, FIR filters with Hamming sinc window were applied to EEG. Firstly, a high-pass filter, with a cut-off frequency at 1 Hz, was applied to remove the DC component and slow-wave drifts. Secondly, a low-pass filter, with a cut-off frequency of 90 Hz was considered, since it is recognized as the upper limit of the frequency band of interest for the analysis. Additionally, a notch filter was also applied in order to remove the powerline interference at 50 Hz.

#### 3.3.2. Channels Spatial Interpolation

After filtering the data, it is important to perform visual inspection of the EEG data in the time domain, and proceed to the removal and replacement of flat or noisy channels (due to electrode malfunctioning), by interpolated signals based on the remaining channels’ information. This interpolation step was performed using the spherical spline interpolation algorithm from Perrin et al. [62].

#### 3.3.3. Re-Referencing

In this study, for the re-reference, the average reference was used, which is performed by doing the average of all 60 channels and the linear transformation of the data. This step is important, not only to eliminate some noise common to all channels and to reduce lateralization bias, but also because of the fact that a reference electrode should not be close or over regions of interest with important brain activity for the analysis [63]. Since the most activated regions during code comprehension are also being investigated, it is important to change the original reference, usually placed between Cz and Pz electrodes, for a proper spatial analysis.

#### 3.3.4. Blind Source Separation

Despite the various preprocessing steps already taken, there are still many artifacts to remove from the EEG signals, such as ocular (eye blinks and eye movement), muscle, and cardiac artifacts. Therefore, independent component analysis (ICA) was applied for blind source separation (BSS) in order to accomplish artifact removal.

**Figure 4:**
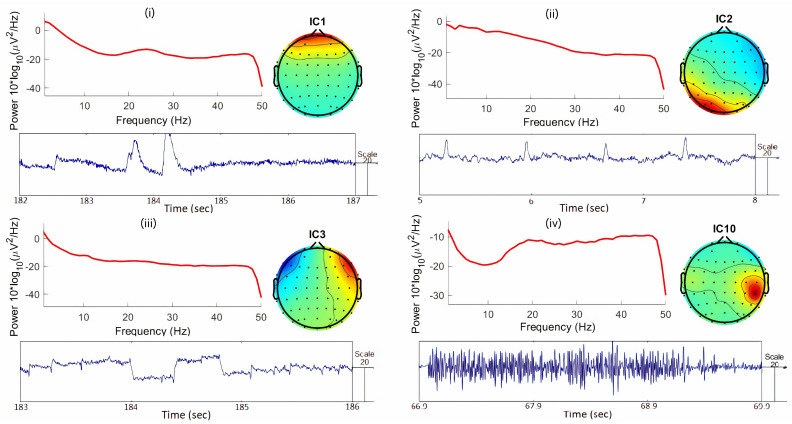
Examples of some artifact components that were identified and removed: (**i**) In component IC1, an eye blinking artifact component can easily be recognized; (**ii**) component IC2 contains cardiac artifact; (**iii**) component IC3 shows another example of ocular artifacts, the saccades/microsaccades; and (**iv**) component IC10 contains involuntary muscle movement.

When preparing the data to run ICA, large muscular activity or other strange events (non-stationary data) were rejected manually from the data, in order to improve the ICA decomposition quality [64]. For this study, the Extended Infomax Algorithm [65] was used for BSS, due to its higher performance in removing ocular and myogenic artifacts [66]. After computing the ICA components, the components associated with artifacts were selected (examples in Figure 4), and subsequently removed, by inspection of the following components: (i) Topographic map; (ii) activity power spectrum; and (iii) continuous time course.

### 3.4. Feature Extraction

After preprocessing the EEG data, a handcrafted feature engineering approach was followed using the most commonly reported features in emotion recognition and cognitive workload literature.

A 1-second window with 80% overlap was used to extract and explore linear features. This type of features are computed using methods that extract amplitude and frequency information from a single EEG electrode (uni-channel) or from multiple electrodes (multi-channel). These features were divided into three groups:

(a) Uni-channel Time Domain features
  - Statistical features commonly used in EEG analysis [67] to characterize amplitude changes and distribution of the signal over time, such as the mean of raw and normalized signal as a measure of the central tendency; variance as measure of the dispersion; skewness as a measure of the distribution asymmetry; and kurtosis as a measure of the distribution tailedness;
  - Hjorth Parameters—in order to describe the EEG signals, Hjorth [68] derived a set of three parameters (activity, mobility, and complexity), widely used nowadays [69]. Activity measures the variance of the signal’s amplitude, and it was already included in the statistical features. Mobility measures the variance of the signal derivative in relation to the variance of the signal’s amplitude. Finally, complexity measures the deviation of the signal from the pure signal with the sine shape.
(b) Uni-channel Frequency Domain features
  Frequency content has long been correlated to cognitive states and, therefore, these features are commonly applied in EEG related analysis. The power spectrum density (PSD) was calculated by squaring the absolute value of the fast fourier transform of the signal. Then, from the PSD, several features were extracted aiming at analyzing specific frequency bands. Among these features it is expected that Theta, Alpha, Beta, and Gamma bands stand out as result of the increase of cognitive workload [54,56,57], either individually or when combined;
  - Power features obtained by computing the area under the PSD curve:
    – Total Power corresponding to the total area of the frequencies of interest.
    – Absolute and Relative Power of frequency bands: Delta (0–4 Hz), Theta (4–8 Hz), Alpha (8–13 Hz), Beta (13–30 Hz), and Gamma (30–90 Hz). The latter given its wide range, was divided into three sub-bands: Low Gamma (30–50 Hz), Medium Gamma (50–70 Hz), and High Gamma (70–90 Hz).
    – Power ratios between bands (combinations of all the seven bands two-by-two), as suggested by [19], it can improve the analysis by minimizing the variability effect of the PSD between subjects.
    – Task engagement indexes, first reported by Pope et al. (1995), are ratios between Theta, Alpha, and Beta power bands, and were being widely used for two possible representative indexes of the participants’ engagement during tasks [70,71,72]:
      (1) $$\mathrm{Index}1=\frac{\beta Power}{\theta Power+\alpha Power}$$
      (2) $$\mathrm{Index}2=\frac{\theta Power}{\beta Power+\alpha Power}$$
  - Average frequency as estimation of the mean frequency of the PSD, in order to explore what are the most predominant frequencies;
  - Alpha peak frequency defined as the frequency corresponding to the maximum peak in the Alpha band has been shown to be able to differentiate mental states [73], with some studies suggesting that it is positively correlated with cognitive performance [74].
(c) Multi-channel features
  - Differential Asymmetry and Rational Asymmetry, defined as the difference and quotient of the power of the frequency bands between pairs of electrodes (left-right brain hemispheres) has been extensively explored to find relations between brain locations [69];
  - Cognitive load index “Brainbeat” was showed by Holm et al. [75] to be a powerful feature in estimating cognitive load during tasks through the ratio between powers of two frequency bands from frontal and parietal brain location:
    (3) $$“\mathrm{Brainbeat}”\;\mathrm{Index}=\frac{\theta Power(Fz)}{\alpha Power(Pz)}$$

Taking into account that the applied setup uses 60 EEG channels, we extracted 480 uni-channel time domain features (8 features × 60 EEG channels), 2400 uni-channel frequency domain features (40 features × 60 EEG channels), and 127 multi-channel features. Thus, a total of 3007 features were computed for analysis.

### 3.5. Feature Normalization

**Figure 5:**
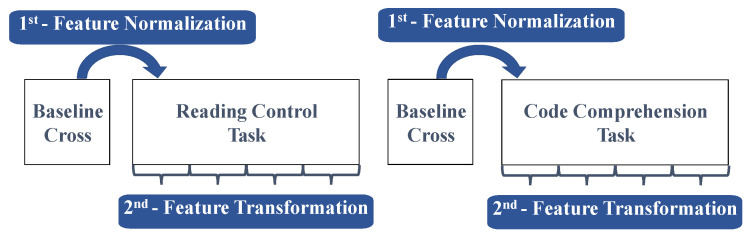
Schematic representation of the feature normalization and transformation steps.

After feature extraction, each feature value was normalized in order to reduce the high inter-subject variability and even to reduce the intra-subject variability throughout the experiments [76]. More specifically, regardin intra-subject variability, this normalization step is crucial, even in small duration experiments, to try to minimize the influence of eventual external factors such as fatigue. Taking into account that there are neutral load events (e.g., the event of the cross), it can be possible to normalize the features with respect to that baseline event. Thus, each cross-event was used for the normalization of the baseline of the next event, i.e., first cross normalized the reading task while the second cross normalized the code comprehension task, as represented in Figure 5. The final value of each feature becomes a variation with respect to the fixation cross baseline task [76]:

(4) $$\Delta {\mathrm{Feature}}_{t,r}\left(k\right)=\frac{Feature_{t,r}\left(k\right)-{\bar{Baseline}}_{t,r}\left(k\right)}{{\bar{Baseline}}_{t,r}\left(k\right)}$$

where *Feature_t,r_*(*k*) is a vector with the values of the feature *k* from the task *t* and run *r*, being normalized by $\bar{Baseline}$_*t*-1,*r*_(*k*), which is the average of the feature *k* in the baseline fixation cross of the same run *r*, and previous to the task *t* being normalized.

### 3.6. Feature Transformation

Following feature normalization, each task was divided into four segments (see Figure 5), and five parameters (second-order features) were computed from the normalized features for each segment of those tasks. The second-order features computed were the following ones: Maximum, minimum, mean, standard deviation, and median. This was performed in order to capture and enhance the state of the subject for each code complexity and respective control tasks, while maintaining sufficient instances for classification.

Concerning multiclass models, in order to differentiate Code 1, Code 2, Code 3, and Control, all the Control tasks were grouped from each trial, as a global Control, and then the maximum, minimum, mean, standard deviation, and median were computed. Thus, at the end there are two final datasets: One with 624 samples (26 subjects × 6 tasks × 4 segments) for binary models and another with 416 samples (26 subjects × 4 tasks × 4 segments) for multiclass models. Both datasets have 15,035 features (3007 features × 5 transformed parameters).

### 3.7. Feature Selection and Reduction

Feature selection and/or dimensionality reduction are of utmost importance, since they might improve the learning efficiency of the classifier, their prediction performance, and reduce the possibility of overfitting [77]. Previously to this step, z-score feature scaling is performed to the data, in order to improve the feature selection or/and classification methods, since the features are all in the same range of values.

In this work, four different approaches were investigated for feature selection and dimensionality reduction, separately.

Three types of rank-based methods were investigated for feature selection with the aim of keeping interpretation regarding the features selected. One of the methods used was the Kruskal–Wallis H test [78] for multiclass classification, or the Mann–Whiney U-test [79] in case of a binary scenario. Another ranking method used was the robust and noise tolerant ReliefF Algorithm [80,81]. On both these methods, the redundant correlated features were eliminated through the computation of the Pearson correlation coefficient [82]. Lastly, the third method explored was the normalized mutual information (NMI) [83], which consists in selecting the best subset of features that besides being relevant, are not redundant [83].

Additionally to the feature selection methods approach previously mentioned, a well-known, widely-used dimensionality reduction technique, the PCA [84], was also tested. The number of principal components selected was based on the percentage of the cumulative explained variance (CEV) that was decided to be maintained [85].

### 3.8. Classification

For classification, we considered four conventional classifiers, in particular, the Fisher linear discriminant analysis (FLDA) classifier [86], the support vector machine (SVM) [87], the k-nearest neighbors algorithm (k-NN) [88], and the Naïve Bayes classifier [89]. We decided to consider a more conservative approach with these classifiers since our main objective was not the classification and finding the best classifier, but instead to demonstrate the separability of the classes.

Although FLDA and SVM are natively binary classifiers, they can be used in multi-class problems by using a one-vs-one, or one-vs-all strategy. Concerning SVM, a linear kernel was used in this study, given its simplicity, speed, and interpretability. Additionally, a linear kernel also reduces the risk of overfitting of our data, which may be more likely if non-linear kernels are used.

The leave-one subject-out cross-validation procedure [90] was considered in this study. This method consists in training the classifiers with the samples of 25 subjects and testing them with the samples of one subject. The choice of this type of cross-validation makes possible to find a robust model that is close to a real application, i.e., classifying new independent samples, from a new different subject, with previous information about other subjects.

The grid search of the parameters in the different feature selection/reduction-classifier models was performed considering a nested leave-one subject out cross-validation. Therefore, the tuned models obtained based on the validation dataset were tested in the independent test dataset, i.e., in the samples of the subject that was left out in each run. For evaluating the performance of the models (feature selection/dimensionality reduction methods combined with different classifiers), we considered five classic evaluation metrics: Recall, precision, specificity, F-measure, and accuracy. In the following section, the classification results achieved for the different tuned models will be presented and discussed.

## 4. Results and Discussion

### 4.1. Software Metrics Labeling Analysis

In this study, the first analysis was performed using a data labeling based on the software metrics of the three codes used in the experiments, being Code 1 the less complex and Code 3 the most complex.

**Figure 6:**
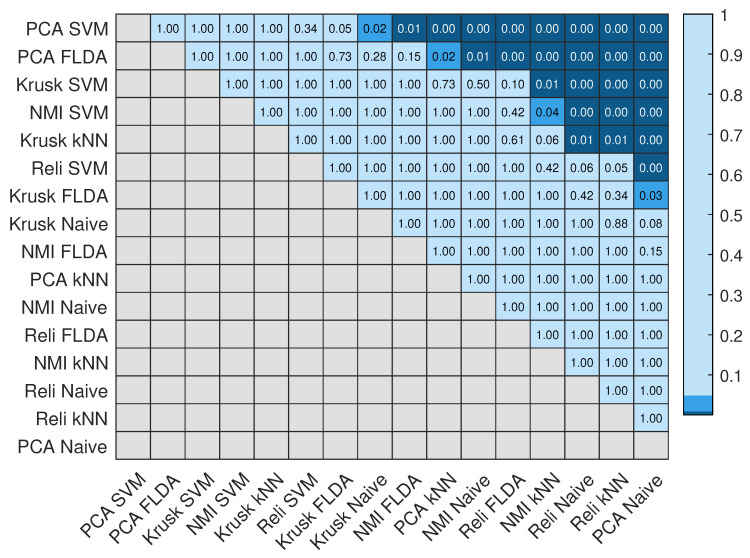
*p*-values of the pairwise comparison of the classification model options, with Bonferroni correction. The values higher than the significance level of 0.05 are colored with light blue, while the intermediate blue is related to a significance level of 0.05 and dark blue to a significance level of 0.01. PCA: Principal Component Analysis; Krusk: Kruskal-Wallis H test; NMI: Normalized Mutual Information; Reli: ReliefF Algorithm; SVM: Support Vector Machine; FLDA: Fisher Linear Discriminant Analysis classifier; kNN: k-nearest neighbors algorithm; Naive: Naive Bayes classifier.

Multiple combinations were explored using the different feature selection/reduction techniques and classifiers, in order to distinguish, through the EEG features, the three code complexities (C1, C2, and C3 labels) and the reading control task (Control label). Afterwards, a statistical analysis aiming to assess differences between the classification models was conducted based on the accuracy results obtained. Figure 6 presents the p-values of the Mann–Whitney test obtained between the different combinations feature selection/reduction-classifier models tuned, considering a significance level of 5% or 1% (different colors). The classification options are ordered from the highest accuracy performance (PCA-SVM) to the lowest accuracy performance (PCA-Naive Bayes).

From these results, it can be observed that the classification options that present more often statistical significant difference from the others, are the ones where the PCA with the SVM or FLDA classifiers were used.

Based on the above, we will present herein the classification results obtained for the example of the option using PCA algorithm and the SVM classifier (see Table 3).

An overall performance of 75% of accuracy was achieved with the linear SVM classifier. The results show a clear distinction of the C1 and the Control tasks from the other two Codes (F-Measure around 94%). However the model is not able to distinguish the more complex codes, i.e., the C2 and C3 (F-Measures only around 50%), which means that from the point of view of the model the participants consider that C2 and C3 have similar complexity.

Another aspect observed is that the F-measure of the reading (Control) task was around 94%. Moreover, by looking at the evaluation parameters, recall and precision, of the C1 and the Control task, it is possible to observe that there are samples of the Code tasks that are being classified as the Control task, whereas all the samples of Control are being classified properly (recall around 99%). Therefore, this was explored by performing a binary classification model of each code comprehension task and the corresponding control task of the same trial (see Table 4). This way, it is possible to verify if the unexpected non-maximum separation of the Control task was due to the normalization step of the classes or instead related to parts of segments with low mental effort during the comprehension of codes in comparison to the mental effort in the reading control tasks, for some participants.

It can be observed that the accuracy results achieved for the classification of C2 vs. Control 2 and C3 vs. Control 3 lie around 100%. However, for the case of C1 vs. Control 1 the results decrease considerably, which might be explained by the low difficulty of the task, suggesting that there are segments during this task that did not present any additional mental effort for some of the participants in comparison to the mental effort in the reading control tasks.

### 4.2. NASA-TLX Labeling Analysis

**Figure 7:**
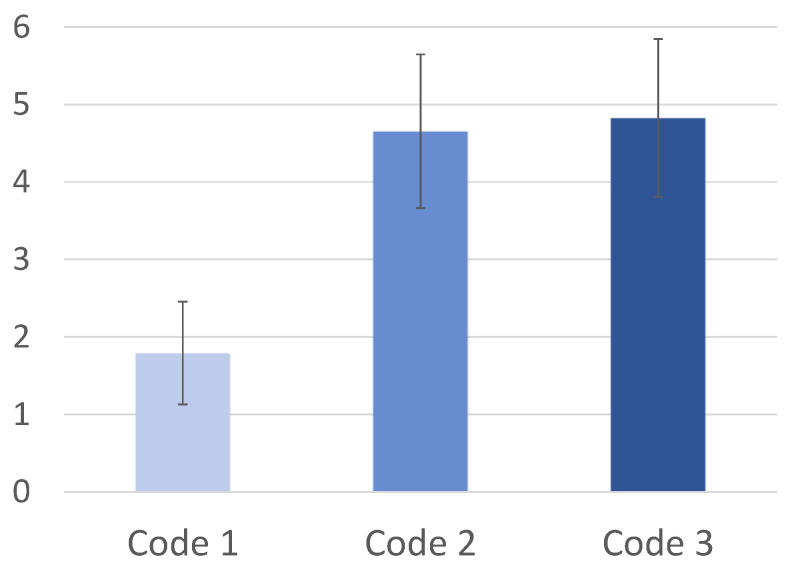
Mental effort felt by the participants and written on the NASA-TLX for the three different Code tasks.

In the previous analyses, the models were designed considering the class labeling according to the software complexity metrics of the code used in the code comprehension tasks. The results obtained reveal that the features discriminate tasks when the code that participants are trying to comprehend have quite different complexity metrics (e.g., C1 vs. C2 and C1 vs. C3). The difficulties in differentiating C2 from C3 suggest a complexity saturation in the participants perception. Therefore, it is possible to conclude that the results do not match the complexity levels evaluated with the software metrics, especially the well-known McCabe Cyclomatic Complexity metric. Nevertheless, the results are coherent with the answers to the NASA-TLX survey, which also points to such saturation behavior in C2 and C3 (see Figure 7).

In view of the results of Figure 7, carrying out a new labeling of the codes’ complexity was considered, according to the cognitive effort of the NASA-TLX. Thus, a new multiclass model was trained in order to distinguish C1, C2/C3, and Control, i.e., two levels of code complexity classes and a control class. The results obtained for this model, which is preceded by the PCA technique and the SVM classifier, are presented in Table 5.

As expected, the overall performance of the model increased with the new labelling, achieving a maximum accuracy of 96.5%. Concerning class C2/C3 classification performance, a F-measure of 99% was achieved whereas the F-measure for Code 1 and Control classes remained similar (around 93%), for the reason already mentioned in the previous results.

### 4.3. Discriminant Features

**Figure 8:**
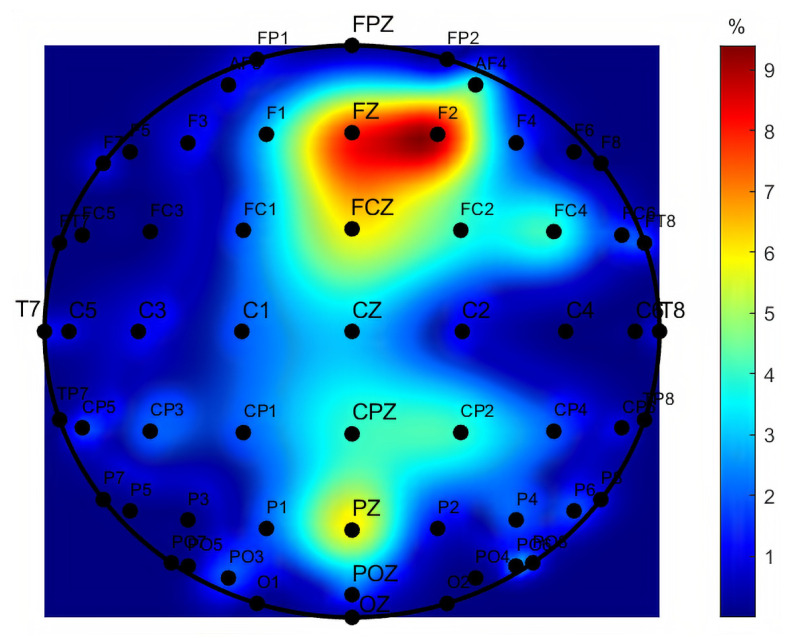
Topographic map representing the percentage of the features corresponding to each electrode after feature selection with Kruskal–Wallis H test, for the multiclass scenario C1 vs. C2 vs. C3 vs. Control.

**Figure 9:**
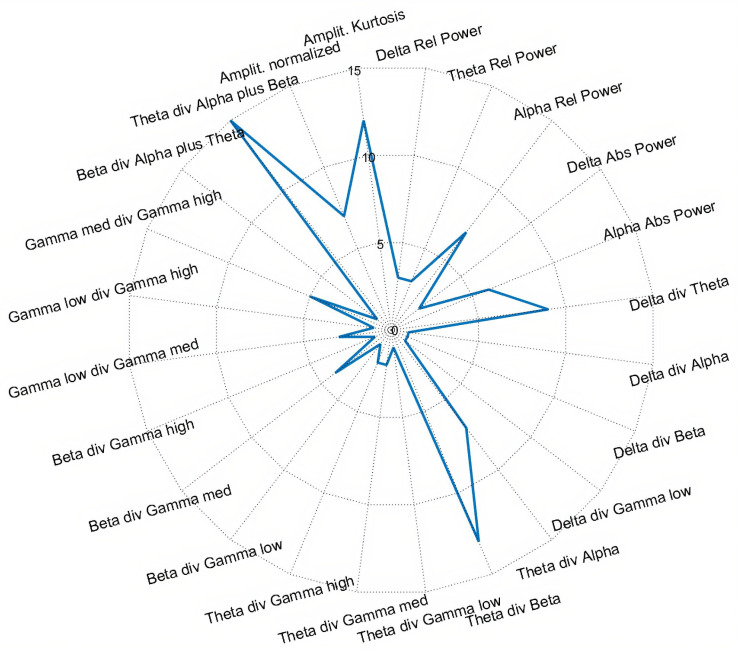
Radar plot depicting which type of features are more frequent (in %) in the dataset obtained after feature selection, for the multiclass scenario C1 vs. C2 vs. C3 vs. Control.

Afterwards, using the Kruskal–Wallis H test as a feature selection method, the most frequent discriminant features were inspected. This was performed by analyzing the first 100 selected features in all folds of validation using the four class labeled dataset (C1, C2, C3, and Control task). Figure 8 presents a topographic map indicating the brain regions corresponding to the most frequent selected features. In Figure 9, a radar plot is depicted containing information about the most frequent type of features selected.

In Figure 8, it is possible to verify that the most frequent features belong to the parietal region (mainly in Pz channel) or near to it, i.e., central parietal (mainly CPz and CP2), and to the evident frontal region (mainly in Fz, F2, and FCz) as well. These results emphasize how important the frontal and parietal electrodes can provide information regarding cognitive workload, going in agreement with the findings of recent studies focused in code comprehension tasks and the mental workload, which pinpoint frontal and parietal regions as the most relevant ones [54]. Furthermore, these findings may potentiate the development of an EEG acquisition system with fewer electrodes, located only on those specific regions, to be used by programmers in software development environments.

Figure 9 shows that the most frequent types of features are mainly related to Theta, Alpha, and Beta band derived features, especially the ratios between them. The type of frequency band features mentioned are in line with the findings of recent studies in the area, which emphasized the significant discriminative power of these bands in distinguishing tasks difficulty and assessing mental workload [54,56,57], suggesting a positive correlation of Theta power and a negative correlation of Alpha power with the increase of code complexity.

### 4.4. Spatial-Temporal Features Analysis

So far, the previous results have been a general analysis of the overall cognitive load associated in the four chunks of the different complexity codes. However, concerning the goal of annotating code lines (or even lexical tokens within each code line) with programmers’ cognitive load, a deeper and more detailed study is needed, exploring the most discriminant EEG features in a spatial-temporal analysis. This was performed by doing a fusion of information with eye tracker data that provides instant information on where the participants are looking, i.e., the gaze points of the eye tracker. This analysis is particularly relevant because it is focused on the accurate assessment of cognitive load while the participants are reading specific code lines. The goal here is to verify if it is possible to determine the exact code lines that require higher cognitive load for a given participant.

**Figure 10:**
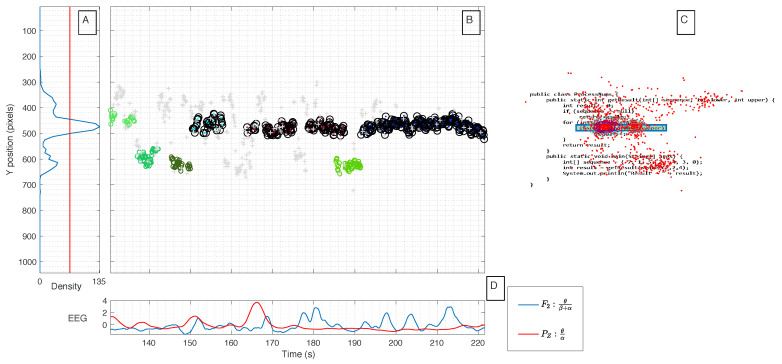
Example of the fusion of EEG with eye tracking, for an intermediate participant during Code task 1. (**A**) Density of gaze points with the red line as a reference of 50% of the maximum row density; (**B**) clusters of gaze points over time and the y-axis; (**C**) code task figure overlapped with gaze points; and (**D**) discriminant EEG features values over time.

As an illustration of what is intended to be performed in this analysis, Figure 10 shows an example of C1 code comprehension for a participant selected at random (almost all participants show a similar behavior).

Regarding eye tracking data, in Figure 10A, it is possible to observe the horizontal density of gaze points along the vertical Y axis of the task. Furthermore, it is also possible to observe the clusters of the gaze points over the experimental time and the Y axis (Figure 10B). The step of clustering was performed using the Density-based spatial clustering algorithm considering a three dimensions feature space (time instants, y-coordinates, and the distance between consecutive gaze points). Finally, the gaze points are overlapped with the code task figure (Figure 10C), where it also represents the geodesic lines that correspond to the clusters with a higher density of gaze points.

**Figure 11:**
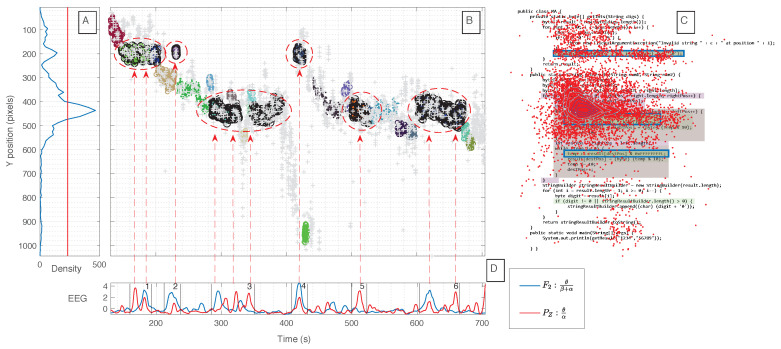
Example of the fusion of EEG with eye tracking, for an expert participant during the Code task 2. (**A**) Density of gaze points, with the red line as a reference of 50% of the maximum row density; (**B**) clusters of gaze points over time and the y-axis; (**C**) code task figure overlapped with gaze points; and (**D**) discriminant EEG features values over time.

In the code task figure (Figure 10C), critical areas of the code (i.e., code lines that are expected to be more difficult to understand by an average programmer) considered by four software developer professionals are also represented. Each developer critical area is marked with a different color, and the common critical areas found by the four developers are marked with a blue rectangle. This can be better visualized in the following example of the Code task 2 (Figure 11).

Finally, the figure also represents two of the most discriminant features from EEG over time (Figure 10D), i.e., the ratio of θ/(β+α) extracted from the electrode $F_{2}$ and the ratio of θ/α extracted from electrode $P_{Z}$. Thereby, by analyzing the variation of the feature values (from only two EEG electrodes) and the corresponding gaze points of the eye tracking data, it makes it possible to achieve a space-time resolution and a deeper and detailed analysis of the cognitive load during the code task under comprehension.

Figure 11 depicts one example for the C2 of one expert participant, which emphasizes the idea of the space-time resolution power in the assessment of the cognitive load over the code, through the combination of EEG biomarkers with eye-tracking information.

From the figure, it can be observed that there are some significant spikes or group of spikes of features (Figure 11D) that match with the clusters with a higher density of gaze points (Figure 11A,B) and with the upper two critical regions marked (Figure 11C), more specifically around the instants: (1) 160–190; (2) 220–240; (3) 280–350; (4) 420; (5) 510; and (6) 600–660 s. From these instants there are spikes that are common to both features, while there are some spikes corresponding to the high density clusters (Figure 11B) or critical regions (Figure 11C) that are only present for one of the features, e.g., the instant represented as (5). This result suggests that depending on the brain region or the frequency band derived features, it can contribute with the same or additional information about the task being performed.

Afterwards, a statistical analysis was performed in order to verify if the values values are significantly different between the critical areas annotated, i.e., code regions that cause higher cognitive load, and non-critical areas. Therefore, based on the eye-tracking information, it was possible to synchronize and select the samples of the EEG features that were inside and outside the critical regions from runs of the participants. After grouping all the data in two classes for each type of feature, we checked the normality of the distributions of the different groups of the two features. Given the groups did not follow a normal distribution, we used the Mann–Whitney U test to evaluate the following null hypothesis (**H0**), and respective alternative hypothesis (**H1**):

**Hypothesis 0** **(H0).** *The proposed EEG biomarkers combined with eye-tracking data can not differentiate code regions that cause higher cognitive load.*

**Hypothesis 1** **(H1).** *The proposed EEG biomarkers combined with eye-tracking data can differentiate the critical regions that cause higher cognitive load.*

We obtained a *p*-value of 0.012 for the feature $F_{2}$:(θ/(β+α)) and a p-value of 0.034 for the feature $P_{Z}$:(θ/α). Considering a significance level α = 0.05, we reject the **H0** and therefore we can conclude that the features values in the regions annotated as difficult to comprehend have statistically significant differences in relation to the features values from the regions not marked as difficult, i.e., we accept the **H1**.

In sum, the overall results of both EEG features, from the two different brain regions highly related with mental workload, reveal possible powerful EEG biomarkers to spot code areas that demand more mental effort or tend to be critical.

The promising findings from this analysis, followed by the results discussed in the previous subsections, support the idea that EEG offers a huge potential in assessing the software programmers cognitive load, in significant space-time resolution, and therefore suggests that future research and applications should focus on using EEG as a reference to validate wearable non-intrusive devices which are more compatible with the software development environment.

### 4.5. EEG as a Reference for Accurate Programmers’ Cognitive State Monitoring

An emergent research area in software engineering and software reliability is the use of wearable biosensors to monitor the cognitive state of software developers during software development tasks.

The EEG biomarkers identified in this work showed enough space-time resolution to allow accurate annotation of code lines with information related to the cognitive state of programmers, opening new possibilities to predict and avoid software bugs. Furthermore, these findings also opens the floor for the usage of the EEG as a reference to validate promising non-intrusive and comfortable wearable devices.

Our proposal is to use the EEG biomarkers and the datasets resulting from this study as a reference to compare, and consequently to validate, other potential biomarkers, particularly biomarkers that can be gathered using wearable non-intrusive devices compatible with the software development activity. This includes biomarkers related to physiologic manifestations driven by the ANS such as heart rate variability (HRV), through wrist-located photoplethysmography (PPG) [91,92], typically done with bracelets or smartwatches, and task-evoked pupillary response [15,93] that is available in most desktop eye tracking systems. Bracelets, smartwatches, and desktop eye trackers are clearly non-intrusive devices fully compatible with software development environments. But the big question is whether this indirect way (i.e., through ANS-related signals) of gathering programmers’ cognitive state information is accurate enough to be used in practice.

The first step to use EEG as a reference to evaluate and improve the accuracy of programmers’ cognitive load gathered using wearable devices is to make our dataset available to the research community. Currently, we have made the dataset available through specific requests to the authors of this study but the dataset will fully be available as soon as this study is published. The dataset includes the following:

(1) Set of programs specifically designed for code comprehension tasks including different complexity levels (measured using the NASA TLX tool) and the annotations made by programming experts identifying the programs snippets that are considered more difficult to understand for the average programmer;
(2) Experiment protocol including the images used as stimulus during the experiment (i.e., the natural language texts, fixation cross, and the software code examples) and the detailed workflow of the experiment to allow reproducibility of the study;
(3) Anonymized raw data collected for the 26 participants (programmers) including EEG and eye tracking data, synchronized and sharing a common time reference. This data is organized in folders per participant, containing for each folder the data of all the trials of code comprehension performed with each participant. The files can be opened using the Matlab EEGLAB toolbox [61].

This set of resources provides an EEG reference of accurate annotation of code lines with information related to the cognitive load of participants while comprehending programs of different complexity. Since it is well known that EEG features present a higher temporal and spatial resolution than features extracted from the ANS-related signals (which are the ones that can be obtained using non-intrusive bracelets/watches, together with desktop eye trackers), the experimental data provided can be used in general to validate future experiments relying on non-intrusive devices. In that sense, the set of resources provided, which includes EEG data, can be used as a reference for future studies.

It is clear that the cognitive load is highly specific for each participant (i.e., different participants with different levels of programming skills may have rather different cognitive loads while comprehending the same code snippet). This is in fact the strongest point of the idea of measuring the (individual) programmer’s cognitive load in order to associate such information to specific code lines for future use as software bug predictor, as the bug predictions will reflect the cognitive state and skills of the actual programmer that wrote/inspected the code. But this also means that care should be taken while using our EEG data to validate other potential biomarkers, particularly biomarkers obtained from ANS-related signals, which are the ones gathered by wearable devices compatible with the software development activity. Since our data includes the characterization of each participant concerning his/her level of expertise, and also includes the results of the surveys done at the end of each trial to determine whether the participant has understood the program or not, it is possible to cluster participants according to their actual level of expertise. Participants of the same level of expertise tend to find similar difficulties in code comprehension, which means that the level of expertise should be used to guide the actual comparison of the results obtained from our EEG biomarkers with other future biomarkers.

## 5. Threats to Validity

Although there are promising results reported in this paper, there are still limitations that will be mentioned and discussed in this section, as the main threats to validity of the present study.

First of all, as the data of this study was acquired in a controlled environment, it will always present limitations regarding the made-up setup, i.e., the natural software environment might not be perfectly realistic, given the natural limits of a complex experiment. Nevertheless, given the limited number of studies using EEG for cognitive load assessment in a software engineering context, our goal here was to provide a systematic analysis using only EEG, by evaluating potential EEG biomarkers present in the cognitive load literature, and proposing ones sensitive to different levels of cognitive load during software activities. Therefore, through the described experiment design protocol, and using carefully designed code samples, the mentioned limitations are not prone to affect the validity of the accomplished results. Moreover, the intra-subject variability of the participants, for example eventual fatigue felt by the participants over the experiment, was carefully minimized by performing the feature normalization of the main tasks in relation to the baseline fixation crosses.

Regarding the code snippets used in the controlled experiment, we are aware that the code snippets could be larger and other software metrics could be used to assess the complexity of the codes. Nevertheless, for practical reasons, we could not use very large programs as the participants would require a considerable amount of time to analyze and comprehend, which would make the experiments unfeasible. We used a task scenario similar to code reviews (focused only on code comprehension) and limited the duration of the experiment for each participant to 45 min. In future work, if the experiment conditions allows, object oriented cognitive complexity metrics should be considered to assess the code snippets.

Another limitation concerns the number of participants (26) in the study. Although 26 participants might be considered a reasonable number for a valid statistical analysis in a complex experiment, we are aware of this limitation and that the number of the dataset should be increased in order to clarify the findings herein described. Additionally, the same goes for the subject group lack of diversity. Despite our effort to gather a balanced group of participants during the screening of participants, unfortunately, the percentage of female software developers (among both Master students and software industry) is quite small when compared to the male percentage, and the group of participants resulted in not being evenly balanced in gender. In future larger datasets, the influence of gender-related factors should be also considered in the analysis.

The last limitation regards the last analysis performed, the spatial-temporal features analysis, since it is an introductory analysis of the feasibility of the potential EEG biomarkers identified in the previous analyses concerning the discriminant features. This type of ambitious analysis, focused on the possibility of determining the exact code lines that require higher cognitive load for a given participant, needs further investigation and validation for establishing the potential code complexity biomarkers as ones to be used in a real space-temporal application in the software industry.

## 6. Conclusions

The controlled experiments performed with 26 programmers showef that EEG could assess programmers’ cognitive load while performing code comprehension tasks with very high confidence. Our results showed that the cognitive load assessed through EEG was consistent with the subjective perception of code complexity measured using the NASA-TLX survey, and both the EEG and NASA-TLX results deviated considerably from complexity metrics in some of the programs used in the experiments. This means that complexity metrics alone are not a good indicator of code complexity as perceived by (human) programmers, which suggests that widespread practice in software engineering such as using metrics to limit complexity of the different modules during software development should be revisited.

Concerning the most discriminant type of features and the scalp regions from which are extracted, the presented results show the importance of Theta, Alpha, and Beta band derived features for the separability between the cognitive load required in low and high complexity code comprehension tasks. In addition, the results also showed that the electrodes on frontal and parietal regions were the ones that most contributed to the discrimination of cognitive load, suggesting that in future applications it might only need a very small set of EEG electrodes in order to have EEG biomarkers as a reference, e.g., using only the two electrodes (F2 and Pz) as presented in the space-temporal features analysis.

More relevant than distinguishing whether a programmer is dealing with complex or simple code is to differentiate exactly the code lines that cause higher cognitive load (or the lines where the programmer may be distracted). This is the key element needed to annotate source code lines with the cognitive load of the programmers while dealing with such code lines (i.e., write, update, inspect), which makes it possible to use such information to improve software quality by providing immediate feedback to the developer on code areas that may need a second look or require a more thorough software inspection or testing. The results of our study showed that EEG biomarkers in association with eye tracking could measure the programmers’ cognitive load in real-time allowing the accurate annotation of the exact code lines where the programmer was looking. This is personalized to each programmer, as the code annotations reflect the way that a specific programmer dealt with the code (e.g., high cognitive load could mean comprehension difficulties but also other states such as distraction could be significant to identify error prone scenarios).

Although EEG can assess programmers’ cognitive load with high accuracy, we consider that EEG is generally too intrusive to be used in normal software development setups. Our conclusion that just two electrodes can be enough to assess cognitive load opens the possibility of using EEG in some specific development scenarios. In any case, we believe that future utilization of programmers’ biofeedback in software development will use non-intrusive wearable devices such as bracelets and watches, together with desktop eye trackers. Since these non-intrusive devices rely on indirect signals driven by the autonomic nervous system, their accuracy in space (i.e., code lines) and time is clearly an issue. The contribution of our EEG study is to provide a reference for future research works, as the high time-space resolution obtained by EEG biomarkers combined with eye tracking can be used to fine tune or validate the results obtained with non-intrusive wearable devices. To allow this utilization as reference we propose to share our experimental setup, including experiment protocol, key elements such as the set of programs with annotations provided by programming experts, the adapted version of the NASA-TLX tool, and the anonymized raw results obtained with the all the experiment participants.

References 

## Figures and Tables

**Table 1:** Brief description of the code complexity software metrics used in the present work.

Code Complexity Metrics	Description
Lines of codes	Measures the number of lines in the code
Number of Parameters	Measures the number of parameters in the code
Weighted Method Count	Measures the sum of the complexity of the code methods
McCabe Cyclomatic Complexity	Measures the number of paths that are linear independent in the code
Nested Block Depth	Measures the maximum nested block depth in the code, i.e., the depth level of the block (e.g., condition) that is deeper in the code

**Table 2:** Electroencephalography (EEG) frequency brain bands and associated brain state.

Name	Frequency Range	Associated State of Brain
Delta	<4 Hz	Unconscious/Deep sleep
Theta	4–8 Hz	Conscious/Imagination/Memory
Alpha	8–13 Hz	Conscious/Relaxed mental activity
Beta	13–30 Hz	Conscious/Emotional/Focused
Gamma	>30 Hz	Conscious/High mental activity

**Table 3:** Multiclass classification results considering software metrics labeling.

Classifier	Multiclass Classification	Evaluation Parameter				
		Recall (%)	Precision (%)	Specificity (%)	F-Measure (%)	Accuracy (%)
SVM (OAO, C = $2^{-10}$)	C1	91.35 ± 19.93	99.23 ± 3.93	99.03 ± 4.73	93.60 ± 14.54	74.76 ± 12.46
	C2	57.69 ± 38.13	51.68 ± 33.39	83.91 ± 12.94	50.60 ± 30.82	
	C3	50.96 ± 36.74	52.06 ± 35.11	85.52 ± 13.70	47.09 ± 30.03	
	Control	99.04 ± 4.90	91.17 ± 14.90	93.75 ± 10.76	94.42 ± 9.67	

**Table 4:** Binary classification results considering software metrics labeling.

Classifier	Binary Classification	Evaluation Parameter				
		Recall (%)	Precision (%)	Specificity (%)	F-Measure (%)	Accuracy (%)
**SVM (C = 2^5^)**	C1 vs. Control	75.96 ± 34.26	73.48 ± 29.98	70.19 ± 36.07	70.67 ± 28.92	73.08 ± 22.27
	C2 vs. Control	100.00 ± 0.00	99.23 ± 3.92	99.04 ± 4.90	99.57 ± 2.18	99.52 ± 2.45
	C3 vs. Control	100.00 ± 0.00	99.23 ± 3.92	99.04 ± 4.90	99.57 ± 2.18	99.52 ± 2.45

**Table 5:** Multiclass classification results considering NASA-TLX labeling.

Classifier	Multiclass Classification	Evaluation Parameter				
		Recall (%)	Precision (%)	Specificity (%)	F-Measure (%)	Accuracy (%)
**SVM (OAO, C = 2^5^)**	C1	91.00 ± 20.26	98.40 ± 5.54	99.33 ± 2.31	92.90 ± 14.74	96.50 ± 5.13
	C2/C3	98.50 ± 4.15	100.00 ± 0.00	100.00 ± 0.00	99.20 ± 2.21	
	Control	98.00 ± 6.92	91.62 ± 13.39	96.00 ± 6.85	93.94 ± 8.30	

## Data Availability

The data regarding the dataset, experiment protocol, and the sample programs are publicly available in the repository of the H2020 project AI4EI (A European AI On Demand Platform and Ecosystem) in the following link: https://ai4eu.dei.uc.pt/base-mental-effort-monitoring-dataset (accessed on 15 March 2021). Any other data can be requested directly from the corresponding author.
